# Nanomedicine for increasing the oral bioavailability of cancer treatments

**DOI:** 10.1186/s12951-021-01100-2

**Published:** 2021-10-30

**Authors:** Alessandro Parodi, Polina Buzaeva, Daria Nigovora, Alexey Baldin, Dmitry Kostyushev, Vladimir Chulanov, Lyudmila V. Savvateeva, Andrey A. Zamyatnin

**Affiliations:** 1 Institute of Molecular Medicine, Sechenov First Moscow State Medical University, 119991 Moscow, Russia; 2 Sirius University of Science and Technology, 1 Olympic Ave, 354340 Sochi, Russia; 3 Belozersky Institute of Physico-Chemical Biology, Lomonosov Moscow State University, 119992 Moscow, Russia; 4 National Medical Research Center of Tuberculosis and Infectious Diseases, Ministry of Health, 127994 Moscow, Russia; 5 Department of Infectious Diseases, Sechenov University, 119991 Moscow, Russia; 6 Faculty of Health and Medical Sciences, University of Surrey, Guildford, GU2 7X UK

**Keywords:** Oral nanomedicine, Cancer treatment, Biological barriers

## Abstract

**Abstract:**

Oral administration is an appealing route of delivering cancer treatments. However, the gastrointestinal tract is characterized by specific and efficient physical, chemical, and biological barriers that decrease the bioavailability of medications, including chemotherapeutics. In recent decades, the fields of material science and nanomedicine have generated several delivery platforms with high potential for overcoming multiple barriers associated to oral administration. This review describes the properties of several nanodelivery systems that improve the bioavailability of orally administered therapeutics, highlighting their advantages and disadvantages in generating successful anticancer oral nanomedicines.

**Graphical Abstract:**

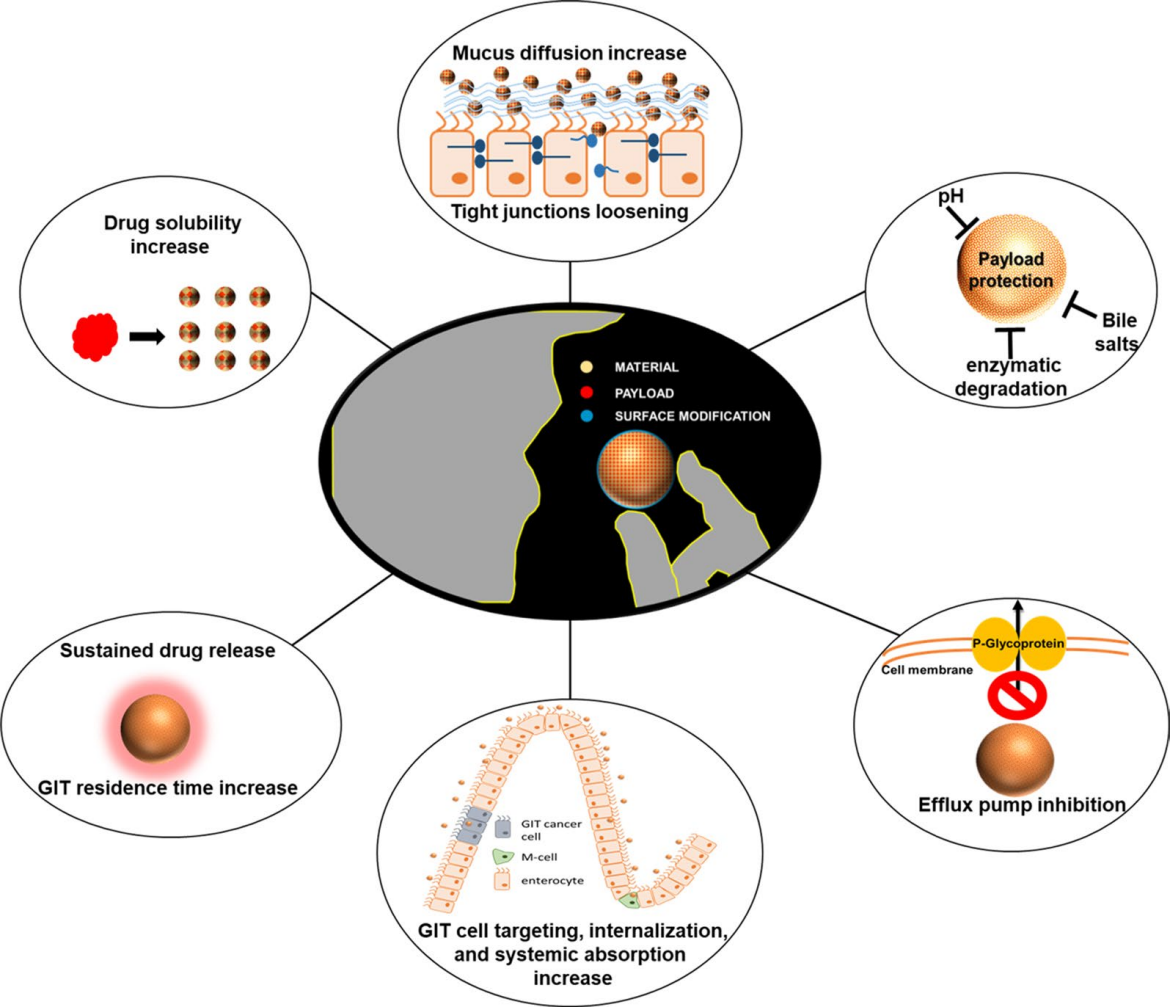

## Introduction

**Fig. 1:**
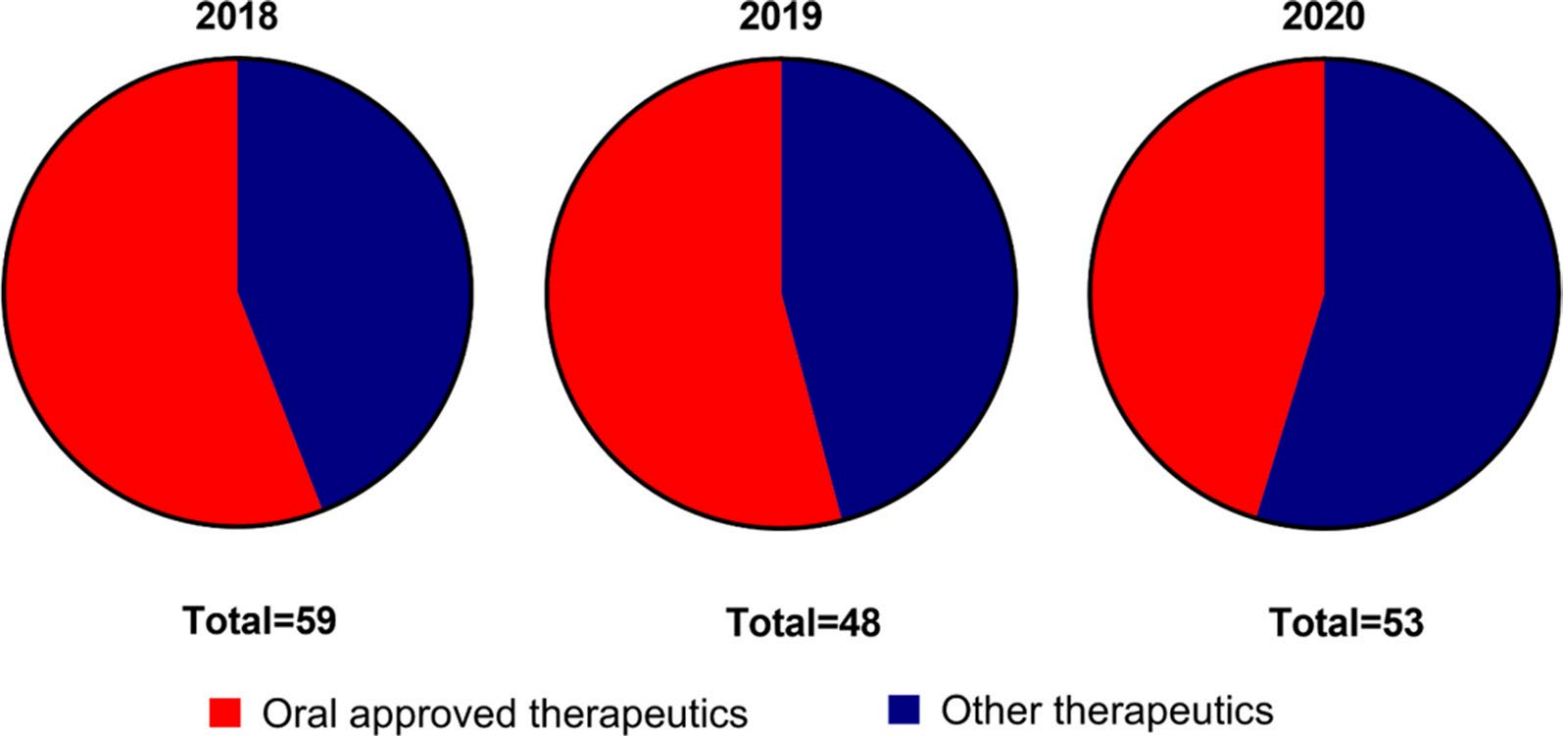
FDA-approved therapeutics in 2018–2020. The graph shows the fraction of approved therapeutics designed for oral delivery (red) compared to therapeutics developed for other administration routes (blue). Source: www.fda.gov

The primary factor determining drug pharmacokinetics and proper concentration at the target site is the administration route. Despite known drawbacks like low drug bioavailability, high degradation, and intestinal and hepatic metabolism, oral treatments have always been considered the most convenient way to deliver a pharmaceutic. This clinical dogma is generally applied to any pathological condition, and most of the approved therapeutics on the market are designed for oral administration [1] (Fig. 1). Oral chemotherapy could be a game-changer for improving patients’ condition while allowing regulation of therapeutic doses without significantly impacting off-target tissues [2]. Finally, oral drug administration is perceived more favorably by patients, particularly compared to infusions and other parenteral administration routes that characterize cancer treatment [3].

Some benefits of oral chemotherapy over standard drug administration routes remain to be proven [4], and patient self-medication has some flaws. For example, shortened hospitalization time decreases clinical evaluation of patient condition and unreported side effects may be overlooked [5, 6]. Self-medication can lead to overdose [7], while other issues are connected to individual differences in drug absorption [8]. Finally, oral drugs are more expensive than traditional parenteral formulations, mitigating the economic benefits of reduced hospitalization time. However, over 90% of patients prefer oral therapeutics [9], and oral chemotherapy may improve palliative treatment in the late stages of disease [10], further prolonging patient lifespan. Traditionally, oral drug delivery is more efficient for treatments based on small molecules. High molecular weight molecules and most new biologic therapeutics are poorly suitable for this kind of administration due to multiple and heterogeneous biological barriers characterizing the gastrointestinal tract (GIT). For example, oral bioavailability of intact proteins can vary from 0.1 to 1% of the administered dose [11, 12]. In addition, the GIT is particularly efficient at decreasing bioavailability of chemotherapeutics, since these molecules are usually affected by low solubility, inactivation under acidic conditions, and low diffusion across cell membranes of GIT epithelium. Also, chemotherapeutics are often substrates of gastric and hepatic metabolic enzymes [13] and transporters like P-glycoprotein (P-gp), which further decrease their bioavailability [14]. New advances in material science paved the way to increase the repertoire of orally administered cancer therapeutics. In particular, nanomedicine and the vast number of carriers for it [15] show solid potential to enhance oral drug delivery of small and high molecular weight molecules. Orally administered nanocarriers can protect the payload from unfavorable biological and chemical conditions of the GIT while favoring controlled drug release. Moreover, oral nanocarriers also enhance targeting towards specific GIT cellular phenotypes, diffuse through the mucous layer, and increase drug bioavailability, avoiding first-pass metabolism.

This review focuses on nanotechnologies tested for oral chemotherapy, highlighting their characteristics for overcoming specific and multiple GIT biological barriers. Some of these barriers also need to be addressed when treating GIT tumors, common and lethal diseases affecting nearly every section of this organ system. Before describing recent examples of oral nanomedicine, a general description of GIT biological barriers and the techniques used to measure intestinal absorption is paramount.

## GIT biological barriers

Different tissue, cellular, physical, and biological components regulate GIT barrier function. The buccal cavity may be an optimal site for drug administration, considering its mildly acidic environment and high submucosal and sublingual space vascularization. However, the transit time of pharmaceutics in the buccal cavity is usually very brief. Therefore GIT remains the primary absorption site for orally administered pharmaceutics [16].

### Physical and chemical barriers

The GIT is a muscular tube 6–10 m long. It is organized into various anatomical regions grouped into 2 two parts: the upper (mouth, pharynx, esophagus, stomach, and duodenum of the small intestine) and the lower section (jejunum and ileum of the small intestine, large intestine, and anus). Nutrient and pharmaceutic absorption varies in the different sections of the GIT. The stomach is the site where the food is broken down and is characterized by small surface area, limited nutrient (and drug) absorption, and very low pH (between 1.5 and 2.5) representing the first chemical barrier encountered by oral pharmaceutics sensitive to acidic pH. However, stomach pH can increase significantly (up to 4–5) after a meal. The small intestine is characterized by slightly acidic pH (5–7.5) that increases in the large intestine (up to 8) [17]. GIT epithelium is lined with mucus, a hydrogel characterized by pores of 200 nm secreted by the goblet cells [18]. Mucus is a physical barrier hampering diffusion of drug molecules into systemic circulation. It is a fluid enriched in negatively charged proteins (mainly mucins) highly modified with proteoglycans [19, 20]. Mucus plays a significant role in facilitating the passage of food through the GIT and protecting tissues from pathogens and low pH [21]. Mucus is quickly turned over and can vary in thickness (200–800 μm) [22, 23], being thickest in the stomach and large intestine (colon), and thinner in the small intestine [24]. Transit time in different parts of the GIT can also significantly affect drug bioavailability, varying between a few minutes to several hours in the stomach depending on whether the person is fasted or fed. Transit time in the small intestine is typically a few hours, while in the large intestine, it can vary from 6 h to a several days [25]. Transit time also depends on age, gender, health state, and food intake amount of the patient. Moreover, mucus composition, transit time, and intestinal fluid composition are strongly affected by the quality and quantity of ingested food and water. All these factors can affect oral drug delivery.

### Biological barriers

Enzymatic degradation is another significant biochemical barrier, particularly for biologics. Pepsin, a broad-range protease, is the main enzyme of the stomach. However, other digestive enzymes, like lipases, are also secreted, and in general, enzymatic composition varies in different GIT sections. In the small intestine, pancreatic and hepatic enzymes break down carbohydrates and nucleic acids in addition to proteins and fatty acids. Mucus is also necessary to provide the ideal environment for enteric microflora proliferation and survival that can change with age, diet, GIT location, and pathological conditions (dysbiosis) [26]. From a therapeutic perspective, the microbiome and the associated enzymatic pathways can represent an additional biochemical barrier. Understanding the different mechanisms occurring during GIT absorption is fundamental for selecting carrier material and surface properties, particularly when the payload needs protection in this environment.

The main factors that govern GIT absorption are the surface area of the tissue, tissue vascularization, water solubility, physical state (suspension, solid, or solution), and concentration of the pharmaceutical at different absorbing sites, [27]. In terms of pharmacokinetics, oral administration does not allow for fast and efficient therapeutic absorption because absorbed drug molecules are affected by the first-pass metabolism that occurs in the intestine and especially in the liver, where drugs are transported through the portal vein after reaching circulation [28]. However, some therapeutics, known as pro-drugs [29], are designed to be enzymatically activated in these sites, and can be utilized to achieve proper drug efficacy. While the stomach has low absorption capacity limited to hydrophobic and non-polar drugs [17], the small intestine is the primary absorption site of the GIT [30]. The surface area of the small intestine is very high due to tissue villi and enterocyte microvilli. Additionally, the small intestine is highly vascularized and connected to the lymphatic system. Finally, in the large intestine, waste processing occurs, and absorption is limited to water [31].

The last barrier is the membrane of the cells lining the GIT. Of these, enterocytes are recognized as the main absorption elements. Nutrients and therapeutics can overcome the epithelial barrier in five distinct ways that can be grossly grouped as active and passive transport. Paracellular and transcellular diffusion are considered passive ways of transport, while receptor-mediated, carrier-mediated, and microfold cell (M-cell)-mediated passage are considered active transport. M-cells, along with enterocytes and some goblet cells, compose the follicle-associate epithelium covering Peyer’s patches, lymphoid structures that are the primary immune system components of the GIT. M-cells have strong engulfment potential and sort pathogens, bacteria, and viruses destined for the components of the underlying immune system [32, 33]. Notably, much effort has been dedicated to understanding active transport mechanisms to increase absorption of different therapeutics. For example, antiviral valine esters were explicitly designed to exploit peptide transporter-1 and neutral and essential amino acid transporters [34, 35]. The phenotypic variability of GIT epithelium is completed by stem cells forming the crypts of Lieberkühn, which reside in invaginations between villi and constantly renew the epithelial cell populations, and by Paneth cells, which secrete factors essential for microflora maintenance.

**Fig. 2:**
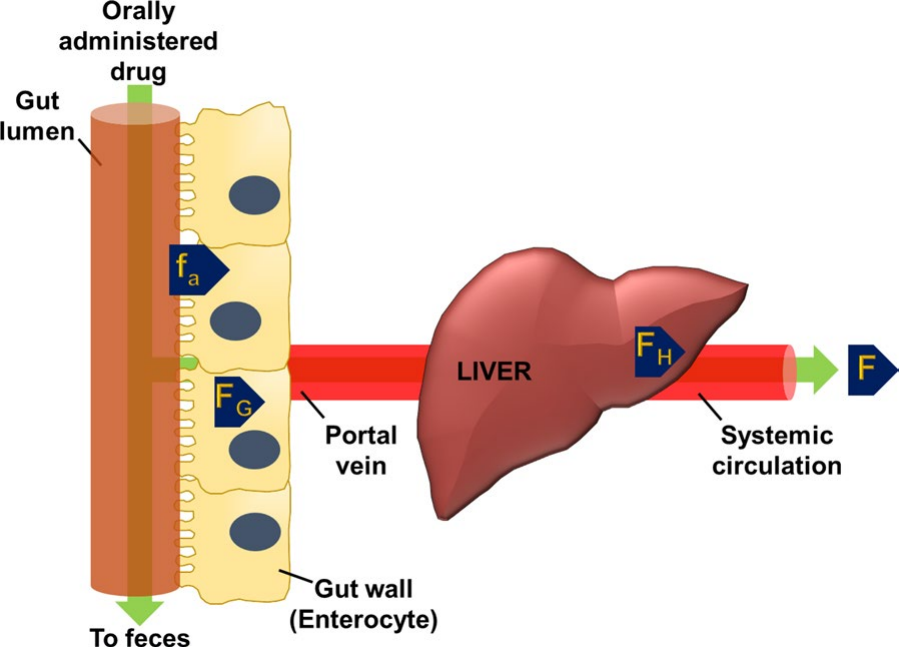
Calculation of the net bioavailability of orally administered therapeutics. Net bioavailability (F) can be extrapolated by calculating the product of the fraction of the drug that overcomes the gastrointestinal tract (GIT) epithelium (f_a_), the fraction of the drug that is not metabolized in the GIT (F_G_), and the fraction of the drug that is not metabolized in the liver (F_H_)

Paracellular transport mechanisms are regulated by epithelial tight junctions, adherens junctions, and desmosomes. The GIT epithelium is permeable only to very small molecules and ions (8–13 Å), with an estimated paracellular cut-off below 2 nm. When the junctions open, this passage can increase to 18 nm, while the underlying layers are impermeable to elements larger than 13–15 nm [36]. Transcellular transport is regulated by the hydrophilic nature of the transported molecule. While hydrosolubility is essential for drug interaction with the GIT epithelium, polarity can inhibit drug diffusion through the cellular membrane. Therefore, to exploit the transcellular pathway, therapeutics must be amphiphilic. According to the Biopharmaceutics Classification Scheme, therapeutics are classified into four different groups as a function of membrane permeability and aqueous solubility [37]. This equilibrium is described by the log *P* value of a molecule, which is a direct measure of its liposolubility. Log *P* is directly related to the molecule's ability to diffuse through the cell membrane and indirectly related to its ability to diffuse in GIT fluids [38]. Net oral bioavailability (F) corresponds to the product of the fraction of the administered dose that overcomes the enterocyte membrane (fa), the fraction that is not metabolized in the gut tissue (Fg), and the fraction that is not metabolized during hepatic first-pass metabolism (Fh) (Fig. 2). Polar molecules can cross cell membranes of enterocytes and M-cells. However, it is worth mentioning that this trafficking can be limited by the expression of transporters like P-gp, multidrug resistance protein 2, and breast cancer resistance protein in GIT epithelial cells, which sort back absorbed molecules.

**Fig. 3:**
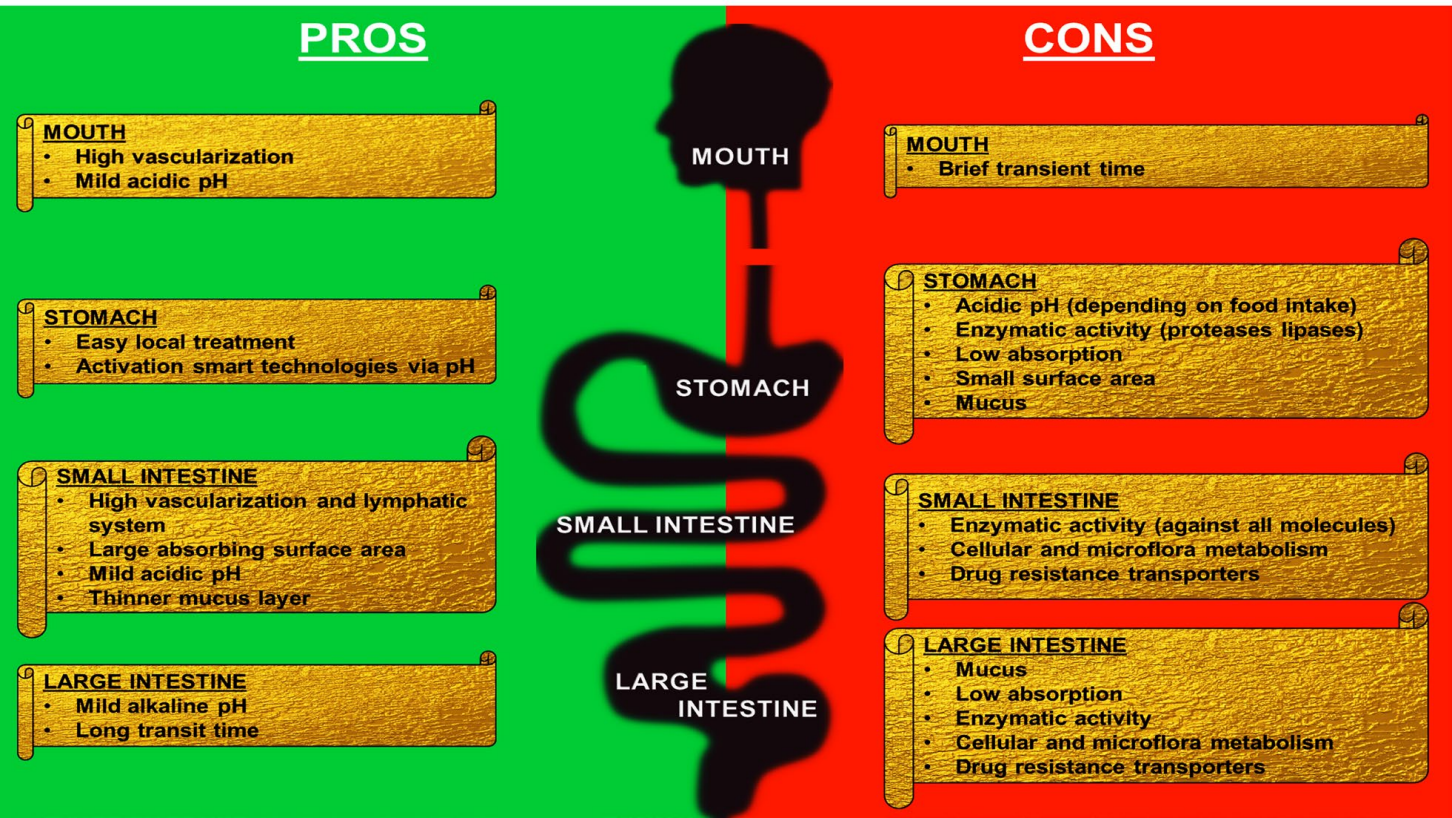
Advantages and disadvantages for pharmaceutical absorption in each GIT section

A general schematic of the major biological barriers hampering oral drug delivery is shown in Fig. 3.

## Experimental models

Different levels of research models are used investigate the absorption of any therapeutic in the GIT environment. Computational models have been generated to predict intestinal drug absorption [39], but unfortunately, only a few attempts were made to apply these tools to oral nanomedicine. To our knowledge, this research field is minimal but growing [40, 41].

### In vitro models

**Fig. 4:**
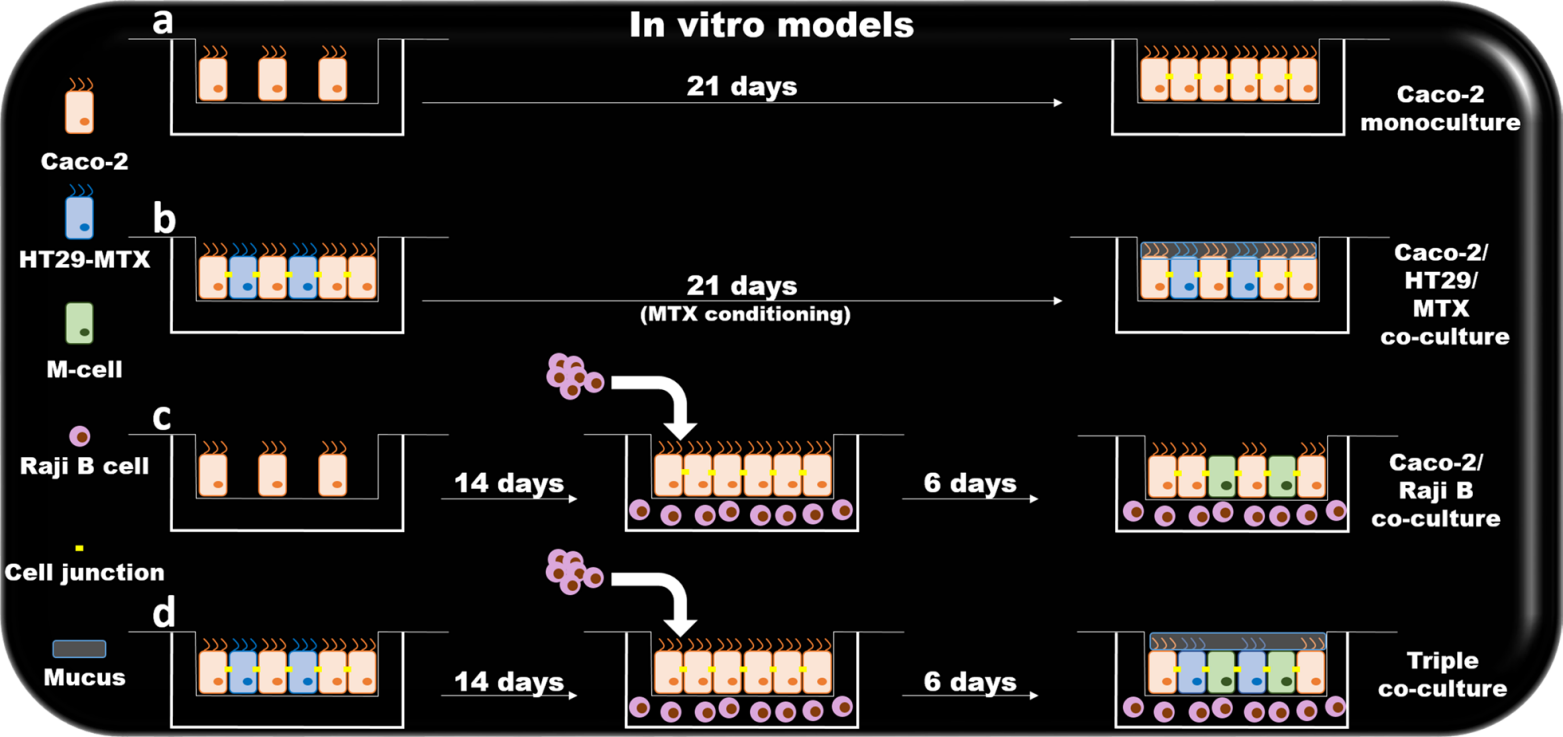
Levels of complexity of in vitro GIT models. **A** Caco-2 cells are widely used for their ability to generate a monolayer characterized by the expression of tight junctions similar to GIT epithelium. **B** Caco-2 cells co-cultured with HT29 cells are induced to secrete mucus. **C** Caco-2 conditioned with Raji B cells can differentiate into M-cells, performing transcytosis. **D** Triple co-cultures allow for the formation of GIT epithelial barrier, mucus secretion, and transcellular transport. (Reprinted from Araújo, F., and Sarmento, B. Towards the characterization of an in vitro triple coculture intestine cell model for permeability studies. 2013. Int J Pharm 15;458(1):128–34. Elsevier.)

Protocols based on simulated intestinal fluids are widely used to investigate the potential of nanomedicine for oral drug delivery. Loaded particles are usually incubated in these buffers to evaluate their stability and drug release properties in the GIT. Simulated GIT fluids are characterized by specific pH, bile salt, and enzyme concentrations to represent the different compartments of the gastrointestinal system. Some procedures describe protocols for generating gastric fluids that simulate fasted and fed conditions, since pH and enzymatic content can vary significantly in these states [42]. The human epithelial colorectal adenocarcinoma cell line Caco-2 is the most common in vitro cellular model of the GIT epithelium. These cells are easy to culture and form microvilli and monolayers connected with tight junctions, as confirmed by the high values of transepithelial electric resistance measurements (260–450 Ω/cm^2^) [43]. Caco-2 cells also express most of the surface markers of the GIT epithelium, including metabolizing enzymes, transporters, and P-gp [44]. Molecular and particle trafficking through this reconstructed in vitro epithelial barrier is usually measured by seeding Caco-2 in transwell systems, though many researchers prefer to co-culture this cell line with other cells like HT29 [45, 46], an adenocarcinoma cell line that can differentiate in mucus-secreting cells after treatment with methotrexate. Transwell systems also allow generation of more complex models by seeding Caco-2 in the upper chamber and lymphocytes isolated from Peyer’s patches of mice in the lower chamber. This cellular conditioning induces Caco-2 to perform transcytosis. A similar effect is obtained by co-culturing Caco-2 with human Burkitt's lymphoma Raji B cells [47] (Fig. 4). From a physiological standpoint, classical in vitro cell culture is not very informative since tissue organization and the vascular, lymphatic, and nervous systems are missing. However, it can provide preliminary data about toxicity on the GIT epithelium and the ability of the therapeutics (and nanoparticles) to overcome the epithelial monolayer and affect its barrier function.

**Fig. 5:**
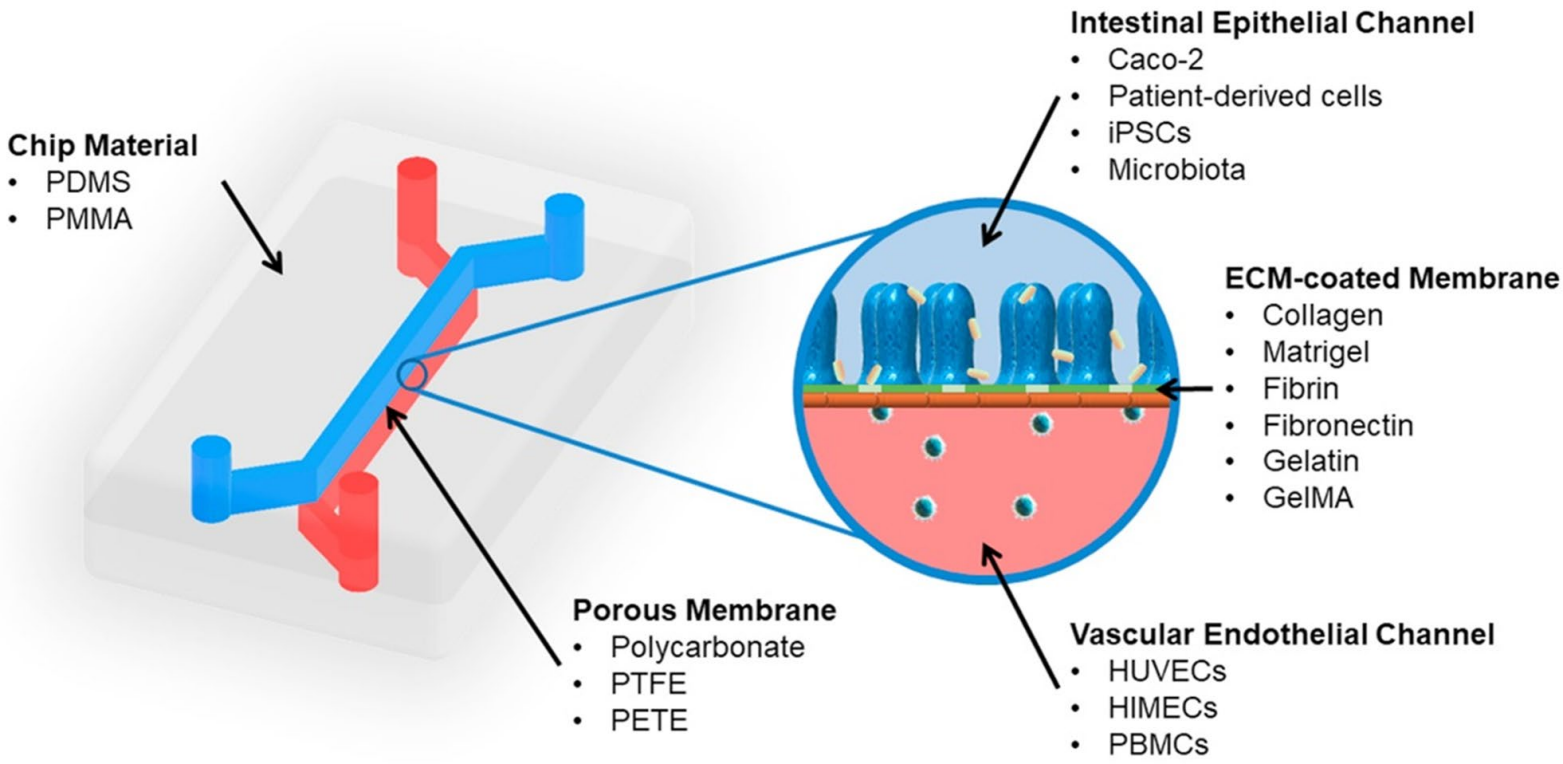
Gut-on-a-chip general schematic. A typical gut-on-a-chip (GoC) is composed of two chambers separated by a semipermeable membrane. One chamber is seeded with cells reconstituting the GIT epithelium, while the second is seeded with other cell phenotypes representing the sub-epithelial environment. (Reprinted from Ashammakhi, N., Nasiri, R., De Barros, N. R., Tebon, P., Thakor, J., Goudie, M., Shamloo, A., Martin, M. G., Khademhosseni, A. 2020. Gut-on-a-chip: Current progress and future opportunities. Biomaterials 225:120,196. Elsevier.)

Organ-on-a-chip systems represent further advances in the field, and in this case, they are referred to as gut-on-a-chip (GoC). In GoC, the goal is to increase system complexity, including elements like microbiota and endothelial and immune cells [48, 49]. Other GIT features like shear and peristaltic forces can also be applied [50]. The basic unit of a GoC is composed of a chamber divided by a semipermeable soft porous membrane into two channels representing the intestinal lumen and the underlying vasculature. The channels can be connected to peristaltic pumps to imitate flow conditions and perform the treatments (Fig. 5) [50]. These systems allow for characterization under optic and fluorescent microscope and for controlling temperature, O_2_, CO_2_, pH, and transepithelial electric resistance (TEER) measurements, and are ideal for investigating epithelial perfusion under more complex conditions [51].

### Ex-vivo, in situ, and in vivo models

Ex-vivo models are based on isolating different sections of the intestine, and barrier integrity and absorption can be investigated in everted [52] and non-everted [53] segments. In the first case, to investigate barrier integrity, molecules or particles of interest are administered outside the tissue that, after eversion, exposes the GIT mucosa. In the second case, instead, the, intestines are ligated and filled with the molecule of interest and absorption is investigated by analyzing the drug content in intestinal tissue. These models preserve the mucus layer and allow simple investigation of paracellular distribution in specific parts of the GIT, particularly in the small intestine, although some damage occurs during tissue preparation. However, from a physiological standpoint, interrupting blood and lymphatic systems and the lack of nervous system components are significant drawbacks of ex vivo systems in pharmacological studies [54].

In situ protocols are performed in vivo by isolating intestinal sections via ligation and locally injecting the molecule of interest. Absorption is then measured in the blood of the subject animal. The physiology of the animal is preserved and absorption can be investigated in specific GIT sections. We recommend the following reviews for more in-depth information of ex situ and in situ procedures [36, 52, 54]. However, these techniques need some surgical and technical skills that can be avoided with classic in vivo experiments in which the therapeutic is administered as a bolus. These protocols totally recapitulate the clinical scenario, since no or minor stress is imparted to the animal during drug administration, and the therapeutics are transported through the whole GIT. New advances in material science have allowed the generation of nanoparticles with increased adherence to the GIT wall. Eventually, accumulation and trafficking of these molecules can be observed through multiple imaging techniques, including in vivo imaging systems and magnetic resonance imaging [55].

### Increasing oral bioavailability through nanomedicine

The use of nanoparticles to enhance oral drug delivery is supported by recent evidence that highlights their versatility and ability to load different therapeutics. Encapsulation can provide a means to increase drug solubility, while particle surface properties can facilitate the penetration of the physical barriers of mucus and epithelial cell membranes. Drug encapsulation also protects the therapeutic payload from the harsh gastric chemical and enzymatic conditions and allows controlled release, which may be helpful for maintaining proper therapeutic concentrations. Finally, nanocarriers allow co-encapsulation of more than one molecule to simultaneously deliver synergistic pharmaceutics that work more precisely together than when administered singularly. Carriers can be generated from biological and organic materials, although inorganic materials and hybrid systems are also routinely tested for this purpose. Each material provides unique features to favor drug encapsulation, protection, and GIT translocation in the bloodstream and the lymphatic system. These features will be described in the following sections, emphasizing the nanoparticle properties that allow negotiation of the GIT biological barriers.

### Polymers

This section refers to organic polymers like polyglycolic acid, polymethylmethacrylate, polylactic acid (PLA), poly(lactic-co-glycolic acid) (PLGA), and poly(ε-caprolactone). These materials have the great advantage of being almost entirely biocompatible and resistant to the GIT environment. Methacrylic co-polymer coating (Eudragit) is commonly used to stabilize oral pharmaceutical formulations like tablets and capsules, and more recently, they have been shown to improve stability of liposomal formulations [56]. For this reason, they are commonly used to generate hybrid technologies to improve oral delivery properties of other materials. However, polymeric nanoparticle synthesis can be expensive, and industrial production can be challenging [57]. PLGA is a frequently investigated class of polymer in this field, and its surface functionalization versatility allows for synthesizing nanoparticles that target specific intestinal transporters, favoring their absorption. For example, when functionalized with carnitine, PLGA nanoparticles can efficiently target Na^+^-coupled organic cation/carnitine transporter 2 (OCTN2), expressed in the lumen of the small intestine, to enhance the delivery of paclitaxel [58]. Characterization of this system and in vitro experiments demonstrated that the success of this kind of targeting depends on several factors. Increasing carnitine concentration on the particles’ surface did not increase their cellular uptake and eventually inhibited their internalization in vitro, demonstrating that optimizing the surface density optimization of the targeting molecule is essential. Secondly, the authors demonstrated that knowledge of the transporter mechanism is necessary to generate efficient oral nanomedicine. In this case, since OCTN2 co-transports carnitine and Na^+^ in a 1:1 ratio, particle uptake increased in the presence of Na^+^. This evidence was confirmed in vivo by administering free and encapsulated paclitaxel via in situ single-pass perfusion. When the therapeutic was administered via PLGA nanoparticles modified with carnitine, higher drug bioavailability was observed; this was also because particle internalization reduced the effect of P-gp, which strongly inhibits paclitaxel. Optimized Na^+^ concentration further improved the process, while free carnitine significantly decreased particle uptake, indicating that these particles must be administered in parallel, with diets containing a certain amount of sodium and no carnitine for reaching optimal therapeutic performance. Finally, the group discovered that much of the absorption occurred in the lymphatic system, suggesting that a certain number of particles can overcome the epithelial barrier via a caveolin-mediated pathway, probably due to OCTN2 receptor recycling. PLA nanoparticles are FDA approved and show high biocompatibility, safety, and sustained drug release [59]. However, when designed for oral administration, they are affected by low GIT absorption, poor mucus penetration, and rapid elimination, though optimizing the surface chemistry of PLA nanoparticles with cell-penetrating peptides [59] can mitigate these drawbacks. Similar benefits are achieved with PLA nanoparticle PEGylation, as demonstrated by the significantly increased bioavailability of encapsulated raloxifene hydrochloride compared to the free administered drug [60], potentially enhancing the usability of this therapeutic for breast cancer prevention [61].

Pharmacokinetic properties of hydrophobic chemotherapeutics like paclitaxel can be improved by modifying PLA particles with folic acid, since the GIT epithelium has significant expression of folate receptors [62]. Folate improved particle diffusion in the mucus layer and particle internalization in GIT epithelial cells. A further modification of this kind of delivery platform with D-alpha-tocopheryl polyethylene glycol (PEG) succinate increased paclitaxel encapsulation efficiency, decreased its release rate, and significantly reduced P-gp drug efflux [63]. These particles were also successfully tested for their efficacy and safety in a rat model of lung cancer.

Polycaprolactone (PCL) is another polymer for generating biocompatible nanoparticles for delivering different chemotherapeutics, including docetaxel [64], cisplatin [65], methotrexate [65], and paclitaxel [66]. PCL nanoparticles have been shown to increase the oral bioavailability of ellagic acid, a natural molecule that has potent anti-cancer, anti-angiogenic, and anti-metastatic activity, but is affected by high hydrophobicity and poor GIT absorption. Encapsulating ellagic acid increased its bioavailability over three-fold by increasing its hydrophilicity and lymphatic absorption via M-cells [67].

### Inorganic materials

Currently, several inorganic materials can be used to generate nanoparticles with different uses and properties. Some of these properties perfectly fit with the scope of engineering nanoparticles for oral administration. Generally, inorganic nanoparticles are considered more stable at body temperatures and acidic conditions, even though some materials are entirely soluble at low pH. Between them, silica nanoparticles were significantly investigated to improve oral delivery of therapeutics. Silica is generally considered biocompatible [68] and easy to modify, and can be made porous to accommodate different payloads. US FDA and European FSA have approved silica as a food and drug additive, and its oral intake is generally considered safe [69]. Amorphous silica has a low dissolution rate at low pH while it degrades faster at higher pH, providing a perfect tool to exploit the GIT pH gradient. Because silica porosity can be easily adjusted, silica nanoparticles can accommodate different payloads [70] including biologics and, after encapsulation, the payload is highly protected from digestive enzymes [71]. Recently, a silica nanostructure was proposed to generate a floating drug delivery system allowing for long gastric retention in the presence of sodium bicarbonate and vegetal polymers [72]. A similar technology based on aluminum silicate was designed to improve the delivery of methotrexate, which has a very short half-life [73]. The benefits of silica for generating oral delivery systems depend on its ability to interact with very hydrophobic molecules to improve their solubility and intestinal absorption due to increased particle internalization while reducing drug efflux phenomena. In vitro, silica did not affect cell membranes and tight junctions of a reconstructed GIT epithelial barrier. Although more toxicological data are necessary, recent observations indicate that silica is better tolerated when orally administered than when it is given intravenously or intraperitoneally.

Another element that can be used for oral nanomedicine is selenium. Its anti-cancer properties are well known, and physiological levels of selenium have high cancer prevention potential [74]. However, clinical selenium use is hampered by significant side effects [75], and the use of selenium nanoparticles is restricted as a supplement for oral administration [76]. Biogenic selenium nanoparticles were synthesized via bacterial production in *Bacillus licheniformis* and tested in vitro for their anti-cancer effects and toxicity. In vivo, compared to commercial selenium supplements, these nanoparticles demonstrated reduced toxicity, probably due to the biological origin of the nanoparticles that increased their biocompatibility. However, when the authors tested the particles in vivo, compared to commercial selenium sources, they saw reduced drug absorption that could partially explain reduced systemic toxicity [76].

### Polysaccharides

**Fig. 6:**
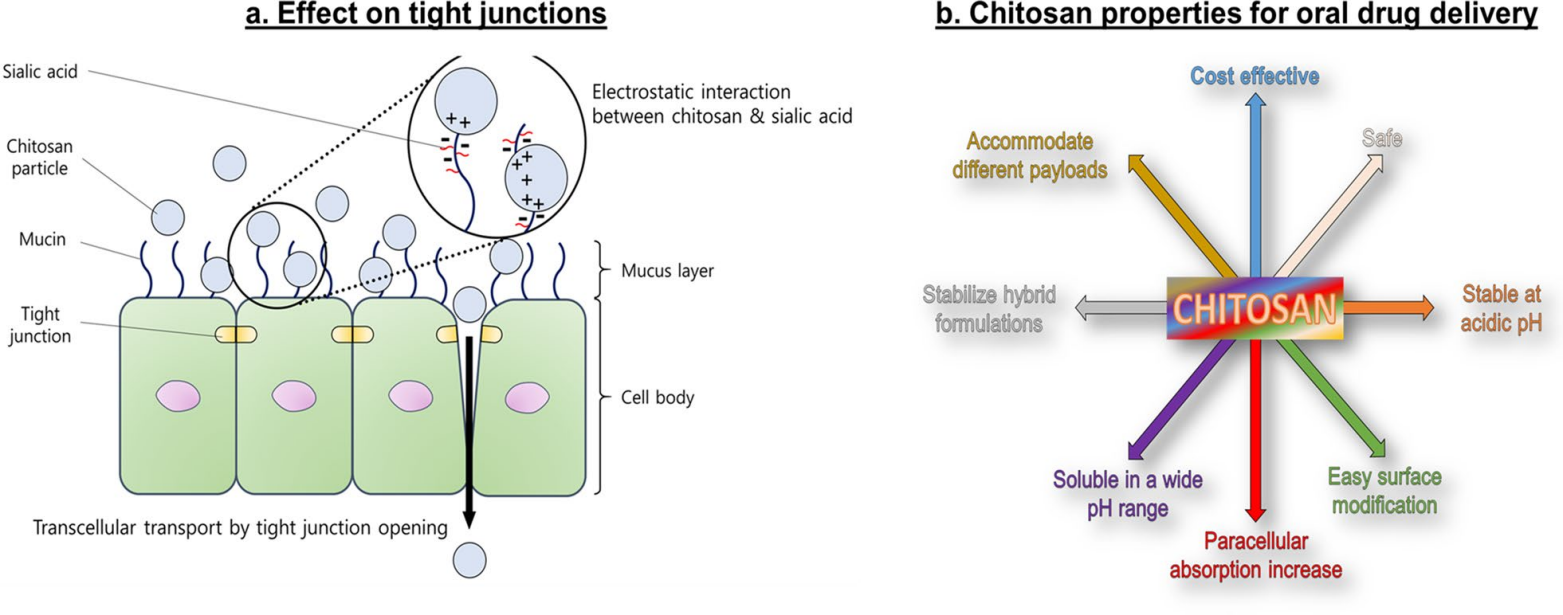
Properties of chitosan in oral nanomedicine. **A** Electrostatic forces between positively charged chitosan nanoparticles and negatively charged GIT epithelial cell surface favor their interaction and eventually lead to tight junction change of conformation that increases transcellular diffusion of particles and their payload. **B** Chitosan properties in generating nanoparticles for oral drug delivery. **A** reprinted from Hong S-C, Yoo S-Y, Kim H, Lee J. 2017. Chitosan-based multifunctional platforms for local delivery of therapeutics. Marine Drugs 15(3):60. MDPI)

Polysaccharides are amphiphilic molecules and provide good wetting properties to overcome the mucus barrier, in addition to their natural ability to encapsulate anti-tumor therapeutics. They are isolated from different biological sources, including animals (chitosan), algae (alginate), plants (pectin), and bacteria (dextran). They are biocompatible, generally easy to manipulate on the nanoscale, and extensively used for pharmacological formulations. As with other nanoformulations, polysaccharide nanoparticles demonstrate the ability to inhibit efflux pump activity [77], and in some cases, they are preferentially absorbed via M-cells [14]. Chitosan represents the gold standard of this class of materials because it was shown to favor tight junction relaxation and increase paracellular absorption [78–80] (Fig. 6a). This phenomenon is most prominent at low pH [81], when chitosan protonation occurs, destabilizing the junctions, but chemical modifications like methylation can extend these properties and increase chitosan solubility in a broader range of pH [82]. Chitosan is also partially soluble in water, generating hydrogel formulations that can accommodate different payloads, including biologics like proteins and siRNA [83]. Chemical modification and hybridization with other materials may make chitosan the most promising material for oral nanomedicine (Fig. 6b). Chitosan nanoparticles modified with acrylonitrile and arginine groups have been shown to enhance curcumin bioavailability [84]. Acrylonitrile provides a hydrophobic skeleton to accommodate this hydrophobic therapeutic while inducing nanoparticle self-assembly, while arginine enhances solubility, increases cell surface interaction, and prolongs residence time and controlled drug release in the GIT [84]. Other hybrid systems based on chitosan modification will be discussed in the following sections.

Another polysaccharide extensively used to generate oral nanomedicine is cyclodextrin, which very efficiently encapsulates hydrophobic drugs (e.g., docetaxel), and can block efflux pump activity [85]. Cyclodextrin nanoparticles significantly increased paclitaxel bioavailability when orally administered, and the drug was detected for 24 h in the bloodstream after administration [86], providing significant benefits in treating a murine model of sarcoma. Similar delivery advantages were observed when orally administered tamoxifen was encapsulated in guar gum nanoparticles [87].

### Protein-based carriers

**Fig. 7:**
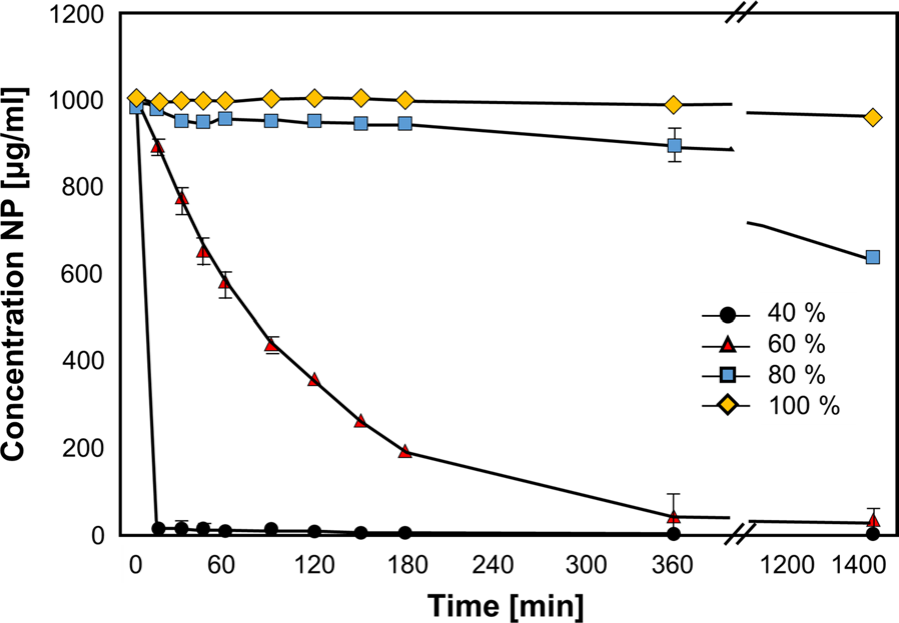
Effects of varying amounts of the cross-linker glutaraldehyde in preventing trypsin degradation of albumin nanoparticles. The figure was reprinted from Langer, K., Anhorn, M.G., Steinhauser, I., Dreis, S., Celeb, D., Schrickel, N., Faust, S., Vogel, V. 2008. Human serum albumin (HSA) nanoparticles: Reproducibility of preparation process and kinetics of enzymatic degradation. Int J Pharm 347(102):109–117. Elsevier

Protein carriers formulated from poly-amino acid chains, gelatin, collagen, casein, and albumin are a few examples of this category. These molecules have amphipathic properties and are ideal for accommodating payloads with different chemical and physical features. M-cells could represent a natural gate for protein nanoparticle transport across the GIT epithelium since they translocate antigens to the underlying lymphoid tissues [88]. Theoretically, protein nanoparticles can easily break down under the action of GIT proteases. The pioneering work of Liu et al. demonstrated that orally administered protein-based pharmaceuticals can be stabilized by co-administering proteases inhibitors [89], even though, to the best of our knowledge, no such protocols have been used to improve cancer treatments. Thus, stabilizing agents like cross-linkers or hybrid formulations were tested for achieving successful drug delivery. Albumin nanoparticles, for example, are extremely sensitive to pepsin and trypsin, but glutaraldehyde cross-linking can protect them from these enzymes [90] and overcome this biological barrier (Fig. 7).

The synthetic protocols used to generate protein nanoparticles can affect their stability against enzymatic degradation. Albumin carriers generated via desolvation are more prone to pepsin degradation than particles synthesized via emulsification, probably due to differential access of pepsin to cleavable peptide bonds [91]. In addition, hybridizing proteins with other materials can increase protein stability in the GIT. For example, coating casein/zein nanoformulations with the carbohydrate pectin was shown to protect the particles while increasing their loading ability (e.g., curcumin) [92].

Moreover, the surface of protein carriers allows multiple sites of chemical modification to increase accumulation of these particles at the intestinal epithelium level, where they can release the payload or, in the case of GIT tumors, improve carrier specificity to target tumor cells. Interestingly, nanoparticles composed of apotransferrin and lactoferrin efficiently increased doxorubicin accumulation in hepatic cancer after oral delivery even though the authors did not investigate the particle/payload absorption mechanism [93]. However, this work is important because it raised doubts about the translational power of current in vivo models, since absorption in rodents may be more efficient than in humans [94].

As in many cases discussed in this review, generation of nanomedicine for oral drug delivery is often driven by the need to enhance oral bioavailability of a therapeutic agent. For example, resveratrol, a hydrophobic polyphenol found in wine, shows significant therapeutic effects in cardiovascular disease treatment and cancer prevention. However, its bioavailability when orally administered is very low (less than 5%) due to its high excretion rate and hepatic and intestinal metabolism [95]. Also, intestinal flora can modify the structure of this molecule via hydrogenation. However, resveratrol can be easily loaded into milk casein nanoparticles via hydrogen and hydrophobic bonds [96, 97]. Casein is a perfect molecule for generating nanomedicine for oral delivery, since this protein is commonly found in food and can be easily engineered at the nanoscale. Penalva et al. [98] demonstrated high efficiency of resveratrol encapsulation in casein nanoparticles, with controlled release of this therapeutic in vitro when the particles were incubated in reconstructed gastric and intestinal fluids. In vivo casein nanoparticles showed excellent propensity to interact and disperse in mucus, increasing in vivo resveratrol bioavailability.

### Hybrid lipid nanoparticles

**Fig. 8:**
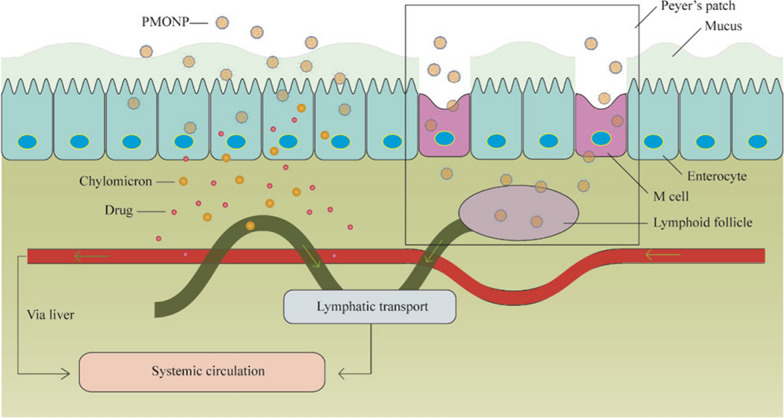
Polymer-lipid hybrid nanoparticle (PMONP) mechanism of absorption. Particle absorption occurs, respectively, via transcellular transport favored by the positive charge of the particles and via M-cell-mediated internalization that increases systemic and lymphatic bioavailability of the payload cabazitaxel. The figure was reprinted from Ren T., Wang Q., Xu Y., Cong L., Gou J., Tao X., Zhang Y., He H., Yin T., Zhang H., Zhang Y., Tang X. 2018. Enhanced oral absorption and anticancer efficacy of cabazitaxel by overcoming intestinal mucus and epithelium barriers using surface polyethylene oxide (PEO) decorated positively charged polymer-lipid hybrid nanoparticles. J. Control Release 269:423–38. Elsevier

Lipid nanoparticles (i.e., liposomes, solid lipid nanoparticles) enhance hydrophobic drug encapsulation solubility, but when used for oral administration, they are often engineered in hybrid formulations to increase their stability in the GIT environment. However, some studies indicate that when interacting with bile salts, liposomes can generate vesicles and micelles that can be absorbed in the upper section of the GIT via transcytosis [99, 100]. Hybrid lipid-polymeric nanoparticles have been designed to increase the oral bioavailability of cabazitaxel [101], which is affected by the typical GIT absorption issues of taxanes (low solubility, high metabolism, P-gp activity). The polymeric structure represented by poly(ε-caprolactone) protect against acidic environment of the stomach, while the lipid component (a medium-chain triglyceride) increases drug loading yield. Finally, a positively charged octadecylamine and neutrally charged polyethylene oxide surface modifications increase mucus layer penetration and cellular uptake, as demonstrated in vitro and in vivo. M-cells probably favor absorption of these particles, since lymphatic transport of cabazitaxel was detected. This approach allows significantly increased oral bioavailability and efficacy of this chemotherapeutic, as shown in vivo in a subcutaneous model of hepatic cancer (Fig. 8).

The group of Dr. Chen developed polymer(pegylated PLGA)-stabilized lipid nanoparticles to increase the oral bioavailability of berberine, a vegetal molecule with numerous therapeutic properties that include anti-neoplastic activity [102]. Oral administration could accelerate its clinical translation, but its chemical structure does not allow efficient absorption. Previous attempts to improve oral bioavailability [103] using liposomal systems failed due to particle degradation in the GIT [102]. For this reason, a PEGylated hybrid lipid-PLGA system was generated to increase particle drug encapsulation, stability, and interaction with the GIT epithelium. The PEG surface modification was crucial to overcoming the mucus barrier and, compared to free drug administration, the authors demonstrated high intestinal absorption in vivo. Similarly, the group of Dr. Cho [104] applied a layer of N-carboxymethyl chitosan to solid lipid nanoparticles that are known to increase the solubility of hydrophobic drugs, provide sustained release of the payload at the pH seen in intestines [105–107], and favor absorption through the lymphatic system, bypassing hepatic first-pass metabolism [106, 108]. N-carboxymethyl chitosan coating increased carrier and payload (curcumin) protection in the stomach environment, eventually favoring its absorption in the mesenteric lymph nodes.

Nucleic acids like short interfering RNAs (siRNA) and long interfering RNAs are a class of biologics that would greatly benefit from oral delivery, but are sensitive to the harsh gastric environment and cannot cross the GIT epithelium [109]. Ball et al. generated particles composed of a lipid mixture, including the amphiphilic lipidoid 306O13 (to complex the RNA), cholesterol, DSPC, and PEGylated lipids, to increase particle stability and mucus penetration of siRNA [110]. They demonstrated that optimal PEG concentration was crucial for successful siRNA delivery across the mucus barrier, confirming the importance of optimizing the density of surface modifications. Despite high delivery efficiency in vitro, stability of these particles was affected by pepsin and bile salts that enhanced their aggregation and degradation, respectively. These data highlighted the importance of analyzing all the factors that compose the GIT environment, since the protease pepsin can still affect the therapeutic efficiency of lipid nanoparticles. In vivo, pepsin concentration in the stomach drastically differs before and after meals, and is reduced during fasting [110]. Testing these conditions, the authors showed higher particle stability and increased small intestine and colon targeting with strong accumulation within intestinal crypts, where the particles could deliver the siRNA to immune cells. This finding highlights the importance of diet and administration time in overcoming GIT barriers.

### Bioinspired systems

In this section, we discuss carriers that can be grown or isolated in nature without extensive bottom-up synthetic procedures and that can be exploited for drug delivery purposes. Of these, microorganisms and exosomes have particular features that are functional for developing oral drug delivery while maintaining precise characteristics on the nanoscale. Microorganisms represent the last frontier for the development of oral nanomedicine since they can specifically target M-cells [111, 112] and potentially be transported to the diseased sites through the lymphatic system [111, 113]. This approach was utilized by the group of Dr. Zhang, who developed a technique to load nanoparticles in yeast and exploit yeast β-glucan capsules to target dectin-1 to M-cells [114]. The external layers and cytoplasm of yeast were chemically eliminated and replaced with therapeutic nanoparticles via electrostatic forces. The carriers were cationic polyethyleneimine loaded with the anti-inflammatory therapeutic indomethacin and paclitaxel; alternatively, iron oxide nanoparticles were used to target the system for magnetic resonance imaging. The authors demonstrated that M-cells mediated transport of yeast loaded with nanoparticles into the lymphatic system, where the particles were recognized and eventually transported to inflamed sites and tumors by macrophages while maintaining significant anti-inflammatory and anti-tumor properties [115].

Microorganisms can also serve as the perfect basis for developing orally administrable cancer vaccines [116]. Cysteine-rich variant-specific surface proteins (VSP) were shown to determine the resistance and colonization of *Giardia lambia* in the GIT tract of mammals. Modifying the surface of retrovirus-like particles with these proteins determined their GIT stability and allowed immunization against influenza virus hemagglutinin (HA). More importantly, this vaccine induced an immune response against a transgenic mesothelioma tumor expressing HA. VSP coating was also shown to increase the immunogenicity of the system, providing a natural adjuvant for immunization. The authors demonstrated that this approach could provide a solid protocol for generating an oral vaccine for cancer when directed against proper antigens [117].

**Fig. 9:**
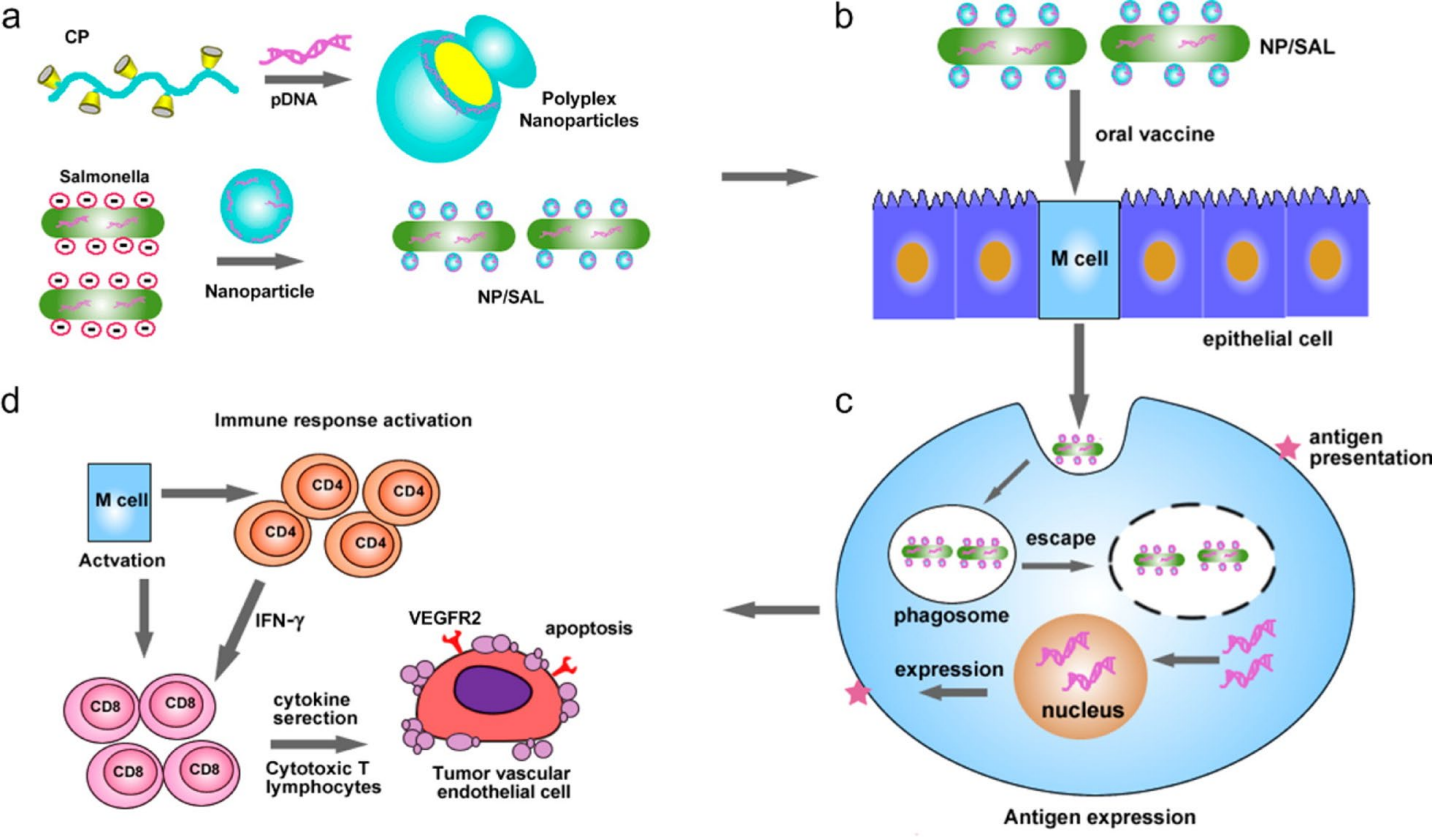
Synthesis and working mechanism of polyplex nanoparticle-coated *Salmonellae*. **A** Polyplex nanoparticles are synthesized by complexing plasmid DNA encoding VEGFR2 with the cationic polymer polyethylenimine and used to coat attenuated Salmonella bacteria. **B** The system is internalized by M-cells and, **C** after endosomal escape, the expression of VEGFR2 on the surface of these cells can **D** induce an anti-tumor immune response via T cell activation. The figure was reprinted from Hu Q., Wu M., Fang C., Cheng C., Zhao M., Fang W., Chu P.K., Ping Y., and Tang G. 2015. Engineering nanoparticle-coated bacteria as oral DNA vaccines for cancer immunotherapy. Nano Lett 15(4):2732–9. ACS Publications

Similarly, live attenuated *Salmonella* bacteria were coated with cationic polymeric nanoparticles condensed with DNA to generate an oral cancer vaccine [118]. The system was designed to be stable at low pH and escape phagosome entrapment after internalization to deliver its immunogenic cargo to the cytoplasm. *Salmonella* bacteria were coated spontaneously via electrostatic interactions between the positively charged nanoparticles and the negatively charged surface of the bacteria. The authors demonstrated that this coating efficiently protected the attenuated bacteria from the gastric environment, favoring their distribution in the blood and the lymphatic tissue. The bacteria also efficiently mediated anti-cancer immunity via T cell activation (CD4 and CD8) and cytokine secretion. Immune system activation occurred due to VEGFR2 antigen expression on phagocytic cells after particle escape from the endosomal compartment (Fig. 9).

Milk exosomes [119–121] possess the necessary attributes for generating safe oral nanomedicine, since these molecules are part of everyday diet, can be easily isolated [120], and are stable at low pH [122]. Milk exosomes showed high efficacy in vitro and allowed for controlled drug (paclitaxel) release in different reconstructed gastric fluids [123]. The nanoformulation of paclitaxel in milk exosomes was stable after several weeks at -80 °C, a fundamental characteristic for its future translational application. In vivo, the loaded exosomes demonstrated significant tumor-killing properties against a subcutaneous lung cancer model, and no adverse effects were correlated with either the carrier or the drug. Interestingly, the authors detected slight tumor growth inhibition upon administration of exosomes only, attributing these effects to some potential anti-tumoral molecules present in the exosome formulation, like complex human milk-derived α-lactalbumin and oleic acid [124]. Even though the absorption mechanism was not completely elucidated, a previous report demonstrated that the exosomes could target different organs, including liver, spleen, kidney, and pancreas, when orally administered. The research in this field is very active and various foods, like grapes, can be viable sources of exosomes, increasing the repertoire of oral nanomedicine platforms [125].

### Nanodelivery systems for the treatment of GIT tumors

GIT tumors are common, often lethal diseases, affecting nearly every section of this organ system. When designing oral nanomedicines for these anatomical sites, it is essential to consider particle stability when targeting the lower GIT and particle adhesion properties when targeting the upper GIT. Oral mucositis is a common condition in cancer patients and often limits the continuity of anticancer therapies, forcing physicians to resolve this issue before continuing other treatments. PLGA nanoparticles were recently developed for treating mouth lesions caused by chemotherapy [126]. They were used to locally deliver rebamipide, a very effective therapeutic against oral mucositis. This drug can be formulated as a mouth wash; however, encapsulation can prolong its residence time in the mouth. The particles were about 100 nm to increase their surface/volume ratio, and they were coated with chitosan hydroxypropyltrimonium chloride to enhance delivery. The positive surface charge of these particles increased their interaction with the mucus in saliva, since mucin proteins are negatively charged. The particles were tested in vitro for their ability to interact with mucin and in vivo for efficacy and increased residence time in the buccal cavity. PLGA nanoparticles have also been designed to deliver docetaxel locally for treating mouth and tongue tumors [127].

The high adhesion properties of polydopamine nanoparticles [128] inspired the generation of a new delivery system to target gastric mucosa and locally delivered the plant-derived anti-cancer molecule xanthatin [129]. This carrier was chosen for its ability to load xanthatin and to adhere to the gastric mucosa. Kotolevets et al. designed a new nanoformulation of paclitaxel in squalene nanoparticles. The synthesis was based on the chemical conjugation of the drug with triterpene and further particle self-assembly, providing a platform with very efficient loading capability [130]. In vitro, the particles demonstrated high cytostatic activity and pro-apoptotic power against different cancer cell lines, including colorectal cancer cells. Also, the particles showed high stability in reconstructed gastric and intestinal fluids with minimal loss of the therapeutic payload. In vivo, orally administered nanoparticles decreased growth of colon cancer compared to free drug administration. The authors showed that drug encapsulation decreased efflux activity of the cancer cells against the therapeutic while increasing its internalization.

Finally, it is worth mentioning the work of Xu et al. [131], who generated oral nanomedicine to revert the stemness of colon cancer, one of the primary causes of its progression and metastatic spread. This system was composed of polymeric nanoparticles (PEGylated PLGA) coated with hyaluronic acid, a polysaccharide shown to efficiently target tumor markers like CD44 and CD168. The particles were loaded with PTC209, an inhibitor of B cell-specific Moloney murine leukemia virus integration site 1; the particles inhibited cancer stem cell proliferation, and showed targeting, efficacy, and safety in vitro and in an in vivo orthotopic model of colon cancer. A list of the nanoplatforms discussed in this section and their properties in increasing the oral bioavailability of anticancer drugs is shown in Table 1.

**Table 1:** Oral nanomedicine for increasing anti-cancer drug bioavailability

	Nanoparticles (NP)	Targeted cancer / tissue	Therapeutic outcomes	Refs.
1. Polymers	Carnitine coated PLGA NP	–	Increased paclitaxel BA	[58]
	Chitosan coated PLGA NP	Oral mucositis induced by chemotherapy	Increased oral cavity residence time	[118]
	PLGA NP	Oral cavity cancer	Increased local docetaxel delivery	[119]
	Pegylated PLA NP	Breast cancer prevention	Increased raloxifene hydrochloride BA	[60, 61]
	Pegylated PLA NP	Lung cancer	Increased paclitaxel BA	[63]
	Hyaluronic acid coated pegylated PLGA NP	Colon cancer	Increased PTC209 delivery and Inhibited cancer stem cell proliferation	[123]
	PCL NP	–	Increased ellagic acid	[67]
2. Inorganic materials	Aluminum silicate	–	Increased methotrexate release properties	[64]
	Selenium NP	Prostate cancer	Reduced side effects and increased tumor growth inhibition	[67]
3. Polysac	Chitosan modified with acrylonitrile and arginine	–	Increased curcumin bioavailability	[74]
	Cyclodextrin micelles	Sarcoma	Increased docetaxel BA and tumor reduction	[76]
	Guar gum (GG) NPs	–	Increased mammary gland targeting, tamoxifen BA, and decreased liver toxicity	[77]
4. Protein	Pectin coated casein/zein NP	–	Enhanced curcumin bioavailability (BA)	[82]
	Apotransferrin and lactoferrin NP	Hepatocellular carcinoma	Enhanced doxorubicin BA and decreased liver nodule number	[83]
	Milk casein NP	–	Increased resveratrol BA than free administered drug	[88]
	Polydopamine NPs	–	Increased gastric targeting and local xanthatin delivery	[120]
5. Lipid nanoparticles	Hybrid lipid- poly(ε-caprolactone) NP	Subcutaneous model of hepatic cancer	Increased cabazitaxel BA and tumor growth inhibition	[92]
	Hybrid polymer-lipid NP	–	Increased berberine bioavailability	[93]
	Chitosan coated solid lipid NP	–	Increased curcumin bioavailability	[95]
	Amphiphilic and pegylated lipids and cholesterol	–	Increased siRNA delivery to immune cells	[101]
	Squalene NP	Colon cancer	Increased paclitaxel delivery and tumor killing properties	[122]
6. Bioinspired systems	Chimeric Virus-like Particles (VLPs) decorated with VSP	HA-expressing tumor	Increased immune response against HA-expressing mesothelioma	[107]
	Live attenuated salmonella coated with polymeric particles	Melanoma	Increased immune response against VEGFR2	[108]
	Yeast loaded polymeric nanoparticles	Subcutaneous breast cancer	Increased paclitaxel delivery via macrophages	[110]
	Milk exosomes	Subcutaneous lung cancer model	Increased tumor killing properties and safety	[115]

## Conclusions

Absorption is the limiting factor for developing efficient oral chemotherapy, particularly for new high molecular weight molecules like biologics [132, 133]. Patient adherence to the treatment poses a significant issue for clinicians. Several initiatives, including smartphone applications [134] and educational programs [135] to increase patient awareness about oral chemotherapy, provided promising results for introducing these therapeutics in the clinic. Many investigations are routinely performed to enhance the absorption of oral formulations. In particular, these studies aim to optimize drug solubility/permeability properties and inhibit P-gp and BCRP transporters [136] using excipients that can eventually favor drug absorption. For example, D-α-tocopherol polyethylene glycol 1000 succinate and PEG-400 were shown to increase the solubility and absorption of etoposide [137], and the research on generating novel, safe solvents is very intense [138]. Nanomedicine for improving oral drug delivery is an emerging field that is a solid option for improving the current administration of chemotherapy and biologics. The scientific community has already identified some materials like chitosan, PLGA, and casein as optimal starting points for success in this field due to their abundance and cost-effective synthesis protocols. Current literature focuses on the advantages of nanomedicine in increasing drug bioavailability, overlooking the potential role of the carriers in increasing drug concentrations at the tumor site. Much work must still be done to define particle limits to overcoming the GIT epithelium, favoring drug release at its interface, and improving tumor targeting. Finally, new evidence shows that, as it occurs in the blood milieu, protein corona derived from gastric fluids and digested food can occur on orally administered nanodelivery systems, potentially affecting their targeting properties and colloidal stability [139].

## Data Availability

Not applicable.

## Abbreviations

GIT: Gastrointestinal tract
GoC: Gut-on-a-chip
PLGA: Poly(lactic-co-glycolic acid)
OCTN2: Organic cation/carnitine transporter 2
P-gp: P-glycoprotein
PEG: Polyethylene glycol
HA: Hemagglutinin
